# Matched comparison of corneal higher order aberrations induced by SMILE to femtosecond assisted LASIK and to PRK in correcting moderate and high myopia: 3.00mm vs. 6.00mm

**DOI:** 10.1186/s12886-021-01987-3

**Published:** 2021-05-15

**Authors:** Mohammad Miraftab, Hassan Hashemi, Mohammadreza Aghamirsalim, Shiva Fayyaz, Soheila Asgari

**Affiliations:** 1 Noor Ophthalmology Research Center, Noor Eye Hospital, No. 96 Esfandiar Blvd., Vali’asr Ave, Tehran, Iran; 2 Translational Ophthalmology Research Center, Tehran University of Medical Sciences, Tehran, Iran

**Keywords:** SMILE, femto-LASIK, PRK, corneal higher order aberrations, dilated pupil, non-dilated pupil

## Abstract

**Background:**

The refractive surgeries induce corneal higher order aberrations (C-HOAs). In this study, change of C-HOAs after small-incision lenticule extraction (SMILE) compared to femtosecond assisted laser in situ keratomileusis (femto-LASIK), and to photorefractive keratectomy with mitomycin-C (PRK) under photopic and mesopic conditions.

**Methods:**

In this prospective study, age, gender, and apical corneal thickness (ACT) matched cases with moderate myopia [spherical equivalent (SE) 3.00 to 6.00D) to high myopia (SE > 6.00D)] were enrolled. In addition to visual acuity and refraction, total C-HOA, coma, spherical aberration (SA), and trefoil in the 3- and 6-mm zones were measured before and 3 and 6 months after surgery.

**Results:**

Overall, 372 moderate myopia cases (124 eyes of 124 individuals in each surgical group) and 171 high myopia cases (57 eyes of 57 individuals in each surgical group) were enrolled. At baseline, the differences in age, gender, ACT, uncorrected and corrected visual acuity, and SE were not statistically significant between subgroups of surgical methods within each myopia group (all *P* > 0.05). At 12 months, in the moderate myopia group, there was less increase in 6-mm zone total C-HOA, coma, and SA with SMILE compared to the other groups (all *P* < 0.05). In the high myopia group, there was greater increase in photopic total C-HOA and trefoil and less increase in mesopic SA with SMILE (all *P* < 0.05).

**Conclusions:**

In correction of moderate myopia, SMILE has better results in mesopic condition. In high myopia correction, femto-LASIK and PRK have better results in photopic and SMILE in mesopic condition.

## Background

Small-incision lenticule extraction (SMILE) which is a flapless procedure for the correction of myopia was first introduced in 2008 [1]. To date, several studies have shown its visual and refractive outcomes individually or in comparison with laser in situ keratomileusis (LASIK). A systematic review and meta-analysis [2], in which 11 out of 102 published articles comparing the two methods were reviewed, showed that the 3 to 6 month outcomes with the two procedures are not different in terms of spherical equivalent (SE), gain and loss of corrected distance visual acuity (CDVA), or predictability, and they are similar in terms of safety and efficacy.

With regard to postoperative visual quality and aberrations, studies suggest SMILE to be superior or equal to LASIK [3, 4] and inferior to wave-front guided LASIK [5]. Comparison of SMILE with laser-assisted subepithelial keratectomy (LASEK) for mild myopia (SE < 3.0D) suggested SMILE to be the preferred method due to less induced aberrations and better patient satisfaction [6]. However, in these studies, aberrations were not compared under photopic and mesopic conditions. This is while aberrations vary by pupil size [7] and should be considered for a more accurate comparison.

The aim of the present study was compare corneal higher order aberrations (C-HOA) in the 3 and 6 mm zones induced by SMILE compared to two conventional refractive surgical methods (femtosecond-assisted LASIK/ femto-LASIK and photorefractive keratectomy with mitomycin C/ PRK) in moderate and high myopia using a matched design.

## Materials and methods

This prospective cohort study was performed on myopic patients undergoing refractive surgery at Noor Eye Hospital in Tehran, Iran in 2020. Myopia was defined based on manifest refractive spherical equivalent less or equal − 0.5 diopter (D). The moderate myopia was considered as spherical equivalent (SE) 3.00 to 6.00D with refractive astigmatism < 2.0D and high myopia SE > 6.00D with refractive astigmatism < 2.0D. Patients were recruited from myopic cases undergoing refractive surgery. Eligibility criteria for surgery were based on age (≥ 20 years), no sign of ectasia, stable refraction in the past 12 months (a change of ± 0.50 D or less), and residual stromal bed (RSB) thickness RSB + cap > 400 μm for SMILE, not including the epithelium RSB > 300 μm for femto-LASIK, and ≥ 350 μm for PRK). All patients had been advised to stop wearing contact lenses for at least 4 weeks prior to surgery. Inclusion criteria of this study were myopia of 3.0 D or more, refractive astigmatism of 2.0 D or less, and no corneal surgical history. Patients undergoing SMILE were enrolled consecutively For each case in the SMILE subgroup, one case from those undergoing femto-LASIK was matched. This individual matching was repeated between SMILE and PRK cases.

### Matching

Only one eye per individual was enrolled. If both eyes were in the same myopia group, one eye was randomly selected and if not, the high myopic eye was selected because of the smaller sample size. Patients in each moderate and high myopia groups were matched in terms of age, gender, and corneal thickness. Matching was based on a range of ± 3.0 years for age and ± 5.0 μm for corneal thickness.

### Ethical considerations

This protocol of this study was reviewed and approved by the Ethics Committee of Tehran University of Medical Sciences (ID: IR.TUMS.MEDICINE.REC.1399.193). Written informed consent was obtained from patients to participate in the study. The study adhered to the tenets of the Helsinki Declaration at all stages.

### Surgical techniques

#### SMILE

SMILE was performed using the VisuMax laser platform (Carl Zeiss Meditec AG, Jena, Germany). After topical anesthesia, patients were asked to fix their gaze on an internal light source. First, the posterior surface of the lenticule was cut from the periphery to the center, and then the anterior surface was cut from the center to the periphery. The parameters for lenticule creation were: cap thickness = 120 μm, cap diameter = 7.7 mm, incision angle = 52◦, incision width = 3.0 mm, optical zone = 6.5 mm, and transition zone = 0.1 mm for cases of moderate myopia, and cap thickness = 120 μm, cap diameter = 7.2 mm, incision angle = 52◦, incision width = 3.0 mm, optical zone = 6.0 mm, transition zone = 0.1 mm for high myopia. The postoperative treatment regimen included chloramphenicol eye drop 0.5 % (Sina Darou, Tehran, Iran) every 6 h for 3 days, betamethasone eye drop 0.1 % (Sina Darou, Tehran, Iran) every 6 h for 1 week, and preservative free artificial tears (Hypromellose) every 6 h for 1 month.

#### Femto-LASIK

For Femto-LASIK, after inducing topical anesthesia, first a 110 μm thick flap was created using Femto LDV (Ziemer Ophthalmic Systems AG, Port, Switzerland). Then the flap was lifted, and wave-front optimized ablation was performed using WaveLight Allegretto EX500 (Alcon, Fort Worth, TX, US) in the 6.50mm optical zone for moderate myopia and 6.00mm for high myopia patients with a blend zone of 1.25mm. The postoperative treatment regimen included chloramphenicol 0.5 % every 6 h for 3 days and betamethasone 0.1 % every 6 h for 7 days.

#### PRK

For PRK, first the corneal epithelium was mechanically scraped without alcohol. Then the WaveLight Allegretto EX500 (Alcon, TX, US) excimer laser was used to free-aberration ablate the 6.50mm optical zone for moderate myopia and the 6.00mm optical zone for high myopia with a 1.25mm blend zone. After laser treatment, a sponge soaked in 0.02 % Mitomycin-C was applied to the ablated stroma for 10 s per corrected diopter. After rinsing with 30 cc sterile balanced salt solution, a bandage contact lens (Ciba vision, Duluth, GA) was applied. The postoperative treatment regimen included betamethasone 0.1 % four times a day, levofloxacin eye drop 5 mg / ml four times a day, and artificial tears as needed. Daily examinations continued until observation of complete epithelial healing. The bandage contact lens was removed upon reepithelialization, and levofloxacin was discontinued; betamethasone and artificial tears were continued for another 2 weeks, after which fluorometholone 0.1 % drops (Sina Darou, Tehran, Iran) was prescribed to be tapered over a course of 3 months.

### Pre- and post-operative examinations

Total C-HOA, coma, spherical aberration (SA), and trefoil (3rd to 7th order) were exported in 3 and 6 mm zones (to simulate photopic and mesopic conditions, respectively) using Sirius (Costruzione Strumenti Oftalmici, Florence, Italy) by a single technician before and 3 and 12 months after surgery. The patient was seated in a dark room for 10–20 min to dilate the pupil at least 6 mm; the pupil size was measured using a pupilometer (Colvard; Oasis Medical, London, UK).

In addition to C-HOA, uncorrected and corrected distance visual acuity (UDVA and CDVA) were measured using the Snellen SC-2000 system (Nidek Inc., Tokyo, Japan), and refraction was determined using retinoscopy (ParaStop HEINE BETA 200; HEINE Optotechnik, Herrsching, Germany).

### Statistical analysis

Analyses were performed using SPSS version 21 (IBM Corp., Armonk, NY, USA). Multiple generalized estimating equations (GEE) were used to examine and compare the 12-month changes in C-HOA indices between the 3 studied groups. Given the individual matched design, unstructured correlation matrix was used in correlation analysis between groups. The significant level was 0.05. Refractive surgery safety index was calculated as postoperative CDVA / preoperative CDVA and efficacy was calculated as postoperative UDVA / preoperative CDVA.

## Results

A total of 543 (372 cases with moderate myopia and 171 cases with high myopia) were enrolled into the study. All surgeries were done by two clinicians (MM and HH) with same experience. No complications were observed during and after the procedures. Demographic information and vision and refraction parameters in the two myopia groups are summarized in Table 1 for SMILE, femto-LASIK, and PRK subgroups. Within each myopia group, the differences between the surgical subgroups were not significant in terms of the parameters listed in Table 1 (all *P* > 0.05), except the difference between maximum lenticule thickness in SMILE and ablation depths in femto-LASIK (*P* < 0.001) and PRK (*P* < 0.001) procedures.

**Table 1:** Demographic information and study parameters in moderate (*n* = 372 eyes) and high (*n* = 171 eyes) myopic patients treated with SMILE, femto-LASIK, and PRK in this study

		Moderate myopia			High myopia		
		SMILE	femto-LASIK	PRK	SMILE	femto-LASIK	PRK
Number of eyes		124	124	124	57	57	57
Age (years)		28.02 ± 5.22	28.21 ± 4.71	28.31 ± 6.32	27.76 ± 6.39	30.29 ± 7.63	28.79 ± 7.00
Sex (F)		66.1 %	62.9 %	64.5 %	61.8 %	58.8 %	63.6 %
ACT (µm)		561.08 ± 25.88	561.03 ± 25.88	560.73 ± 25.87	544.56 ± 17.26	551.00 ± 16.50	543.62 ± 18.66
Removal tissue thickness (µm)		106.63 ± 13.51	76.43 ± 13.79	73.41 ± 13.61	143.53 ± 16.28	112.33 ± 11.63	112.56 ± 12.38
MRSE (D)	Pre-op	-4.66 ± 0.85	-4.47 ± 0.82	-4.36 ± 0.72	-7.54 ± 0.92	-7.19 ± 0.61	-7.79 ± 1.37
	After 12 M	0.15 ± 0.34	-0.00 ± 0.33	0.10 ± 0.35	-0.05 ± 0.47	-0.31 ± 0.28	-0.27 ± 0.58
Astigmatism (D)	Pre-op	-1.00 ± 0.80	-0.80 ± 0.58	-0.93 ± 0.71	-1.64 ± 1.15	-1.56 ± 1.41	-1.51 ± 0.91
	After 12 M	-0.40 ± 0.40	-0.39 ± 0.29	-0.45 ± 0.29	-0.57 ± 0.53	-0.50 ± 0.05	-0.37 ± 0.20
UDVA (logMAR)	Pre-op	1.40 ± 0.26	1.37 ± 0.28	1.40 ± 0.14	1.75 ± 0.15	1.62 ± 0.33	1.73 ± 0.21
	After 12 M	0.01 ± 0.03	0.00 ± 0.00	0.00 ± 0.02	0.01 ± 0.03	0.02 ± 0.06	0.05 ± 0.13
CDVA (logMAR)	Pre-op	0.00 ± 0.00	0.00 ± 0.00	0.00 ± 0.00	0.01 ± 0.03	0.00 ± 0.00	0.02 ± 0.04
	After 12 M	0.00 ± 0.01	0.00 ± 0.00	0.00 ± 0.00	0.00 ± 0.00	0.01 ± 0.02	0.01 ± 0.02
Safety index		0.99 ± 0.03	1.00 ± 0.00	1.00 ± 0.00	1.04 ± 0.07	0.98 ± 0.04	1.04 ± 0.07
Efficacy index		0.99 ± 0.02	1.00 ± 0.00	1.00 ± 0.00	1.04 ± 0.07	0.98 ± 0.04	1.04 ± 0.07

*SMILE *small incision lenticule extraction, *femto-LASIK *femtosecond-assisted laser in-situ keratomileusis, *PRK *photorefractive keratectomy with mitomycin-C, *ACT *apical corneal thickness, *MRSE *manifest refraction spherical equivalent, *UDVA *uncorrected distance visual acuity, *CDVA *corrected distance visual acuity

Removal tissue thickness: maximum lenticule thickness in SMILE and ablation depth in femto-LASIK and PRK

### Total C-HOA

**Fig. 1:**
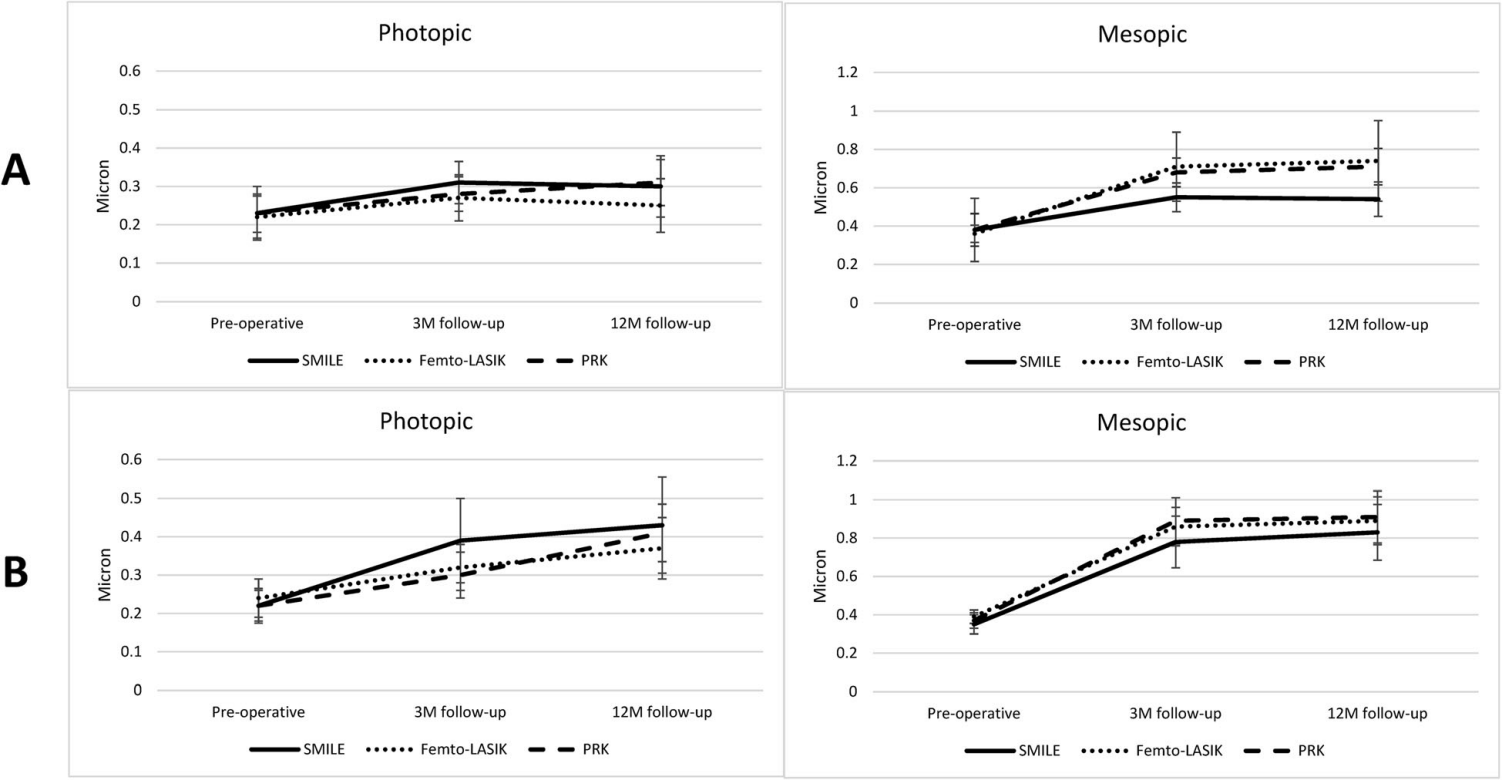
One-year change in corneal higher order aberrations after refractive surgery for moderate (**a**) and high (**b**) myopic patients in the photopic and mesopic conditions. SMILE: small-incision lenticule extraction, femto-LASIK: femtosecond assisted laser in situ keratomileusis, PRK: photorefractive keratectomy with mitomycin-C, M: months

Figure 1 shows one-year change in total C-HOA in the study groups. In moderate myopia group, total C-HOA increased in all 3 subgroups in photopic and mesopic conditions (all *P* < 0.05). The increase in 6mm total C-HOA after SMILE was lower than femto-LASIK (0.16 ± 0.32 vs. 0.38 ± 0.36 μm, *P* < 0.001) and PRK (0.33 ± 0.25 μm, *P* = 0.001). But the changes in 3mm total C-HOA were not significant between SMILE and femto-LASIK (*P* = 0.098) and SMILE and PRK (*P* = 0.184).

In high myopia group, the significant increase in 6mm total C-HOA was not different between SMILE and femto-LASIK (*P* = 0.234) and PRK (*P* = 0.079). But increase in 3mm C-HOA after SMILE (0.21 ± 0.24 μm) was higher than femto-LASIK (0.13 ± 0.17 μm, *P* < 0.001) and PRK (0.19 ± 0.15 μm *P* = 0.004).

### Coma

In the moderate myopia group, 6mm coma increased in the surgical subgroups. The increase with SMILE was less than the femto-LASIK (*P* = 0.012) and PRK (*P* = 0.021). But in 3mm zone, there was no difference between SMILE and two the other procedures (both *P* > 0.05) (Table 2). In the high myopia group, 3mm and 6mm coma increased in three surgical subgroups, but the differences between SMILE and the other subgroups were not significant (all *P* > 0.05) (Table 3).

**Table 2:** Baseline and postoperative 3mm and 6mm higher order aberrations in cases of moderate myopia treated with SMILE, femto-LASIK, and PRK

Index	Pupil diameter	Surgical method	Pre-operative	3 M follow-up	12 M follow-up	One-year change	*P*-value
Total coma (µm)	6-mm	SMILE	0.22 ± 0.21	0.36 ± 0.16	0.38 ± 0.18	0.16 ± 0.23^a^	**0.012**^b^ **0.021**^c^
		Femto-LASIK	0.21 ± 0.09	0.41 ± 0.23	0.46 ± 0.31	0.25 ± 0.23^a^	
		PRK	0.24 ± 0.17	0.45 ± 0.18	0.48 ± 0.20	0.24 ± 0.24^a^	
	3-mm	SMILE	0.11 ± 0.10	0.14 ± 0.08	0.15 ± 0.11	0.04 ± 0.12	0.313^b^ 0.791^c^
		Femto-LASIK	0.17 ± 0.62	0.14 ± 0.11	0.16 ± 0.15	-0.01 ± 0.64	
		PRK	0.11 ± 0.08	0.14 ± 0.09	0.14 ± 0.13	0.03 ± 0.11	
Total SA (µm)	6-mm	SMILE	0.21 ± 0.04	0.30 ± 0.10	0.33 ± 0.12	0.11 ± 0.09^a^	**< 0.001**^a^ **< 0.001**^c^
		Femto-LASIK	0.21 ± 0.05	0.42 ± 0.10	0.45 ± 0.10	0.24 ± 0.10^a^	
		PRK	0.22 ± 0.07	0.43 ± 0.12	0.48 ± 0.11	0.26 ± 0.13^a^	
	3-mm	SMILE	0.07 ± 0.03	0.07 ± 0.04	0.06 ± 0.03	-0.01 ± 0.04	0.125^b^ 0.482^c^
		Femto-LASIK	0.06 ± 0.02	0.07 ± 0.03	0.07 ± 0.04	0.01 ± 0.03	
		PRK	0.07 ± 0.03	0.07 ± 0.04	0.08 ± 0.03	0.01 ± 0.04	
Total trefoil (µm)	6-mm	SMILE	0.14 ± 0.19	0.14 ± 0.07	0.13 ± 0.05	-0.01 ± 0.17	0.198^b^ 0.271^c^
		Femto-LASIK	0.14 ± 0.06	0.18 ± 0.19	0.16 ± 0.15	0.02 ± 0.19	
		PRK	0.14 ± 0.08	0.16 ± 0.08	0.15 ± 0.05	0.01 ± 0.11	
	3-mm	SMILE	0.12 ± 0.09	0.16 ± 0.08	0.14 ± 0.08	0.02 ± 0.12	0.122^b^ 0.342^c^
		Femto-LASIK	0.13 ± 0.09	0.14 ± 0.08	0.13 ± 0.12	0.00 ± 0.11	
		PRK	0.12 ± 0.07	0.15 ± 0.08	0.14 ± 0.10	0.02 ± 0.11	

^a^ Within subgroup statistically significant change

^b^ Comparison of one-year change in indices between SMILE and femto-LASIK

^c^ Comparison of one-year change in indices between SMILE and PRK

*M *months, *SA *spherical aberration, *PRK *photorefractive keratectomy, *SMILE *small incision lenticule extraction, *femto-LASIK *femtosecond-assisted laser in-situ keratomileusis

**Table 3:** Baseline and postoperative 3mm and 6mm higher order aberrations in cases of high myopia treated with SMILE, femto-LASIK, and PRK

Index	Pupil diameter	Surgical method	Pre-operative	3 M follow-up	12 M follow-up	One-year change	*P*-value
Total coma (µm)	6-mm	SMILE	0.20 ± 0.09	0.52 ± 0.27	0.56 ± 0.28	0.36 ± 0.28^a^	0.535^b^ 0.289^c^
		Femto-LASIK	0.21 ± 0.09	0.50 ± 0.22	0.54 ± 0.22	0.33 ± 0.19^a^	
		PRK	0.21 ± 0.10	0.48 ± 0.24	0.52 ± 0.26	0.31 ± 0.24^a^	
	3-mm	SMILE	0.09 ± 0.06	0.14 ± 0.11	0.16 ± 0.12	0.05 ± 0.11^a^	0.233^b^ 0.823^c^
		Femto-LASIK	0.10 ± 0.07	0.16 ± 0.06	0.16 ± 0.09	0.06 ± 0.08^a^	
		PRK	0.09 ± 0.05	0.13 ± 0.08	0.14 ± 0.10	0.05 ± 0.10^a^	
Total SA (µm)	6-mm	SMILE	0.21 ± 0.07	0.46 ± 0.15	0.45 ± 0.15	0.24 ± 0.14^a^	**0.006**^b^ **< 0.001**^c^
		Femto-LASIK	0.23 ± 0.05	0.56 ± 0.12	0.53 ± 0.12	0.30 ± 0.13^a^	
		PRK	0.22 ± 0.06	0.61 ± 0.21	0.60 ± 0.21	0.38 ± 0.21^a^	
	3-mm	SMILE	0.06 ± 0.03	0.06 ± 0.03	0.08 ± 0.04	0.02 ± 0.04	0.106^b^ 0.722^c^
		Femto-LASIK	0.06 ± 0.03	0.08 ± 0.04	0.07 ± 0.03	0.01 ± 0.04	
		PRK	0.07 ± 0.03	0.06 ± 0.05	0.07 ± 0.04	-0.00 ± 0.06	
Total trefoil (µm)	6-mm	SMILE	0.14 ± 0.07	0.16 ± 0.08	0.17 ± 0.08	0.03 ± 0.09	0.426^b^ 0.130^c^
		Femto-LASIK	0.17 ± 0.10	0.22 ± 0.15	0.21 ± 0.16	0.04 ± 0.16	
		PRK	0.13 ± 0.08	0.19 ± 0.11	0.18 ± 0.10	0.05 ± 0.12^a^	
	3-mm	SMILE	0.13 ± 0.09	0.21 ± 0.12	0.23 ± 0.10	0.10 ± 0.15^a^	**< 0.001**^b^ **0.006**^c^
		Femto-LASIK	0.15 ± 0.09	0.16 ± 0.11	0.18 ± 0.13	0.03 ± 0.14	
		PRK	0.13 ± 0.09	0.16 ± 0.09	0.15 ± 0.11	0.02 ± 0.14	

^a^ Within sub-group statistically significant change

^b^ Comparison of one-year change in indices between SMILE and femto-LASIK

^c^ Comparison of one-year change in indices between SMILEand PRK

*M *months, *SA *spherical aberration, *PRK *photorefractive keratectomy, *SMILE *small incision lenticule extraction, *femto-LASIK *femtosecond-assisted laser in-situ keratomileusis

### Spherical aberration

In the moderate myopia group, the significant increase of SA with SMILE was less than the femto-LASIK (*P* < 0.001) and PRK (*P* < 0.001) only in 6mm zone (Table 2). In the high myopia groups, similar to the moderate myopia, 6mm coma was increased with all surgical procedures. But SMILE was associated with lesser increase compared to the other procedures (all *P* < 0.05). The increase of 3mm coma was not different between SMILE and the other two procedures (both *P* > 0.05) (Table 3).

### Trefoil

In the moderate myopia group, within and between change in 6mm trefoil was not different with three procedures (all *P* > 0.05) (Table 2). In the high myopia group, despite of significant increase of 6mm trefoil with PRK, the difference between SMILE and PRK (*P* = 0.426) was not significant. The increase in 3mm trefoil with SMILE was less than the femto-LASIK (*P* < 0.001) and PRK (*P* = 0.006) (Table 3).

## Discussion

### Total C-HOA

The findings of the present study in individuals with moderate myopia (3.0 to 6.0 D) and low astigmatism (< 2.0D) suggest that there is no difference between SMILE and femto-LASIK or between SMILE and PRK in terms of C-HOA induction when assessments are done under photopic conditions (pupil diameter: 3.0mm). However, at a 6.0mm pupil diameter, SMILE is associated with less HOA induction. In cases with high myopia (> 6.0D) and low astigmatism (< 2.0D), there is greater increase in total C-HOA at 3.0mm pupil diameter due to induced trefoil with SMILE than with the other two methods. But at a 6.0mm pupil diameter, there is less SA induction with SMILE than the other two procedures. These results were stabled after 3-month follow-up.

### Coma

Induced coma after surgical procedures can be due to various factors. In SMILE, femto-LASIK, and PRK-MMC, coma can be decentration-induced or develop as a result of asymmetric or irregular wound healing [8–11]. Also, induced coma with SMILE may be due to lack of iris registration [12]. In our study, SMILE induced very small amount of photopic pupil coma (0.04 ± 0.12 μm) in patients with moderate myopia and results were comparable to the other procedures. But under mesopic conditions (6.0mm pupil), there was less coma induced with SMILE which could be due to better centration in SMILE group [13], or maybe the amount of decentration with femto-LASIK and PRK has small impact on visual quality under photopic conditions. But, Yildirim et al. [14] reported no difference between SMILE and aberration-free PRK in terms of induced coma for correction mild and moderate myopia. This difference could be related to severity of myopia. As the pupil dilates and aberrations increase in mesopic conditions, outcomes achieved with SMILE appear to be superior to the other two procedures.

In our high myopic cases, compared to the moderate myopia group, the amount of induced coma was greater with all three approaches. Even in the presence of an eye tracker in excimer laser assisted procedures, the longer procedure time needed for higher correction can contribute to fixation fatigue and decentration-induced aberrations [8, 9, 12]. Regardless of the degree of myopia and pupil size, the amount of coma induced by SMILE was equal to or less than that by the other two methods. Similarly, in the study by Yang et al. [15], induced coma in the myopic SMILE group (0.22 μm) was not significantly different from the femto-LASIK group (0.13 μm).

### Spherical aberration

In refractive surgery, the shape of the cornea is changed from prolate to oblate. The greater the degree of correction and ablation depth, the greater the corneal aspheric change and the greater the SA. Therefore, the amount of induced SA in the high myopia group was significantly higher than the moderate myopia group. The amount of 3.0mm SA induced by the three procedures was not significantly different. However, regardless of the degree of myopia, there was less 6.0 mm SA induction with SMILE. This could be attributed to the fact that the integrity of the anterior corneal layers is better preserved with SMILE. Some studies showed that vertical side cut of corneal lamellae during flap creation in LASIK lead to loss of corneal stiffness and mid peripheral corneal bulging which increase SA [16]. Also, the larger functional optical zone after SMILE was associated with smaller corneal asphericity (more prolate) and may have a contribution to lower SA [17].

### Trefoil

In our high myopia group, SMILE showed greater 3.00mm trefoil induction compared to femto-LASIK and PRK. However, there were no significant differences in the 6mm pupil. This could be due to micro-distortion of the corneal center in the correction of high myopia [18]. In the sample of our study, trefoil increased by 0.10 μm after 12 months and in the study by Yildirim et al. [19] increased by 0.02 μm one year after surgery. Since trefoil impacts the retinal image quality less than other HOA, these observations may be of little clinical value.

In choosing the best surgical method, in addition to eligibility criteria, the patient’s occupation and lifestyle should be considered. Aberrations vary by pupil size which depends on incoming light and working distance [20]. In tasks such as driving at night, maintaining mesopic vision quality is more important, and therefore SMILE can be recommended. In near work, such as surgery, maintaining quality of vision under photopic conditions should be the priority, and thus, femto-LASIK or PRK are recommended.

## Conclusions

As a limitation of the present study, it was not possible to apply random allocation to the three surgical methods due to the patient eligibility criteria of each method. However, since we applied multiple matching, we can conclude that in cases with moderate myopia and low astigmatism, SMILE is preferred to femto-LASIK and PRK because of less induction of HOAs in low light conditions. In cases of high myopia and low astigmatism, femto-LASIK and PRK offer better daylight results while SMILE offers better outcomes in low light conditions. In other words, the choice of surgical method will should be based on the patient’s condition.

## Data Availability

The data will be available in case of reasonable request by corresponding author.

## Abbreviations

SMILE: Small-incision lenticule extraction
femto-LASIK: Femtosecond-assisted laser in situ keratomileusis
PRK: Photorefractive keratectomy with mitomycin C
CDVA: Corrected distance visual acuity
UDVA: Un corrected distance visual acuity
C-HOA: Corneal higher order aberrations
SE: Spherical equivalent
RSB: Residual stromal bed
SA: Spherical aberration
GEE: Generalized estimating equations
